# Numerical Analysis of Mechanical Characteristics of Constant-Resistance, Energy-Absorbing and Anti-Scour Bolts

**DOI:** 10.3390/ma15103464

**Published:** 2022-05-11

**Authors:** Zhi Tang, Hao Wu, Ying Liu, Yishan Pan, Jinguo Lv, Dezhi Chang

**Affiliations:** School of Mechanics and Engineering, Liaoning Technical University, Fuxin 123000, China; wuhao7783843@163.com (H.W.); liuying119966@163.com (Y.L.); panyishan@lnu.edu.cn (Y.P.); glvjinguo2005@163.com (J.L.); changdezhi01@163.com (D.C.)

**Keywords:** constant-resistance energy-absorbing, anti-scour bolt, constant resistance anti-impact device, impact energy, impact velocity

## Abstract

In order to improve the impact resistance mechanical properties of bolt, the requirements of rock burst roadway support must be met. Based on the requirements that the anchor should have a reasonable deformation load threshold, high stroke efficiency, constant reaction force and stable repeatable deformation damage mode. A constant resistance anti-impact device was designed, and a new constant resistance energy-absorbing impact anchor rod was designed in combination with a conventional anchor rod, and the working principle of a constant resistance energy-absorbing impact anchor rod was given. ABAQUS finite element software was used to analyze the mechanical properties of bolt and the results showed that the constant resistance energy-absorbing anti-shock anchor has a stable and repeatable deformation damage mode under both static and impact loads, and the three indexes of the constant resistance energy-absorbing anti-shock anchor, such as yield distance, impact resistance time and energy absorption, are significantly better than those of the conventional anchor. The impact energy and impact velocity have less influence on the load-bearing capacity and stroke efficiency of the impact device. The impact velocity has less influence on the indices of the rod yield load, breaking load, absorbed energy and the yield distance of a conventional anchor and constant resistance energy-absorbing anti-stroke anchor, and the impact resistance time decreases non-linearly with the increase in the impact velocity.

## 1. Introduction

Coal is the main energy source and an important industrial raw material in China. and is the cornerstone of China’s energy security. With the gradual depletion of shallow coal resources, deep coal resources mining has gradually become the new normal for coal resources development. Deep mining, coal mine impact pressure [1,2,3] and other power disasters will be more serious, bringing great harm to the safe and efficient production of coal mines [4,5,6,7]. Statistics show that 90% of impact pressure accidents occur in the roadway, and impact pressure roadway support has become a major problem for safe coal mining.

Anchor support [8,9,10] is a common method in roadway support in China, but the traditional anchor is not designed from the angle of impact prevention, and it is difficult to resist the strong impact load and fail. Pan Yishan et al. [11,12,13] proposed the theory and technology of roadway energy-absorbing yielding impact protection support, and in recent years, practice has proved that the use of energy-absorbing support can effectively prevent roadway impact ground pressure [14,15,16,17].

In recent years, domestic and foreign scholars have conducted a lot of research on energy-absorbing anchors [18,19]. Wang Qi et al. [20] conducted a comparison test of static tension and dynamic impact of conventional anchor rods, new anchor rods and constant resistance energy-absorbing anchor rods, and the results showed that constant resistance energy-absorbing anchor have good impact resistance and overall deformation capacity. Xin Liwei [21] concluded that the single bubble body giving way to energy-absorbing members can help improve the anchor support level in high stress and large deformation roadways. Tao Zhigang [22] revealed the mechanical change law and energy absorption characteristics of NPR anchor/rope in the whole process of surrounding rock control. Fu Yukai et al. [23] analyzed the mechanism of high-impact toughness anchor rod (cable) energy absorption and its application effect. Sun Xiaoming et al. [24] studied the tensile mechanical properties of constant resistance large deformation anchor rods. Li Chen et al. [25] analyzed the mechanical properties of constant-resistance large-deformation anchor rods in terms of force and deformation at different rates of impact. Lian Chuanjie et al. [26] concluded that high-strength prestressed compression anchor rods can reduce the anchor rod load and prevent the anchor rod from prematurely entering into yielding. The results of existing studies show that incorporating energy-absorbing devices with energy-absorbing properties in anchor rod design can effectively absorb the impact energy of the surrounding rock and enhance the mechanical properties of the anchor rod against impact. However, the existing energy-absorbing anchor rods are costly, the constant resistance performance needs to be improved, and the installation generally requires secondary punching. To solve this problem, a new type of constant resistance energy-absorbing anchor rod is designed, and its mechanical properties are analyzed by numerical simulation.

## 2. Design of Constant-Resistance Energy-Absorbing Anti-Scour Anchor

### 2.1. Design of Constant Resistance Anti-Impact Device

The constant resistance anti-impact device should have the following basic characteristics [27,28,29]. (1) It should have a reasonable deformation load threshold. The impact protector does not deform or deform less before the surrounding rock pressure reaches the deformation load threshold. The yield load in the force–displacement curve of the constant resistance anti-impact device is its deformation load threshold, and the deformation load threshold is set at 90–99% of the rod yield load to ensure that the constant resistance anti-impact device only functions when the rod is about to yield. (2) It should have a high stroke efficiency. (3) It should have a more constant reaction force. The reaction force of the constant resistance anti-impact device in the process of being crushed and deformed by the surrounding rock should be kept as constant as possible to ensure that the rod is deformed in yielding after the deformation of the constant resistance anti-impact device is over. (4) It should have stable repeatable deformation damage mode. To ensure the reliability of the constant resistance anti-impact device in complex situations.

**Figure 1:**
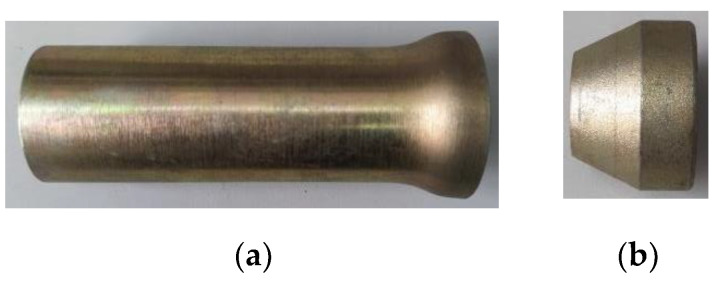

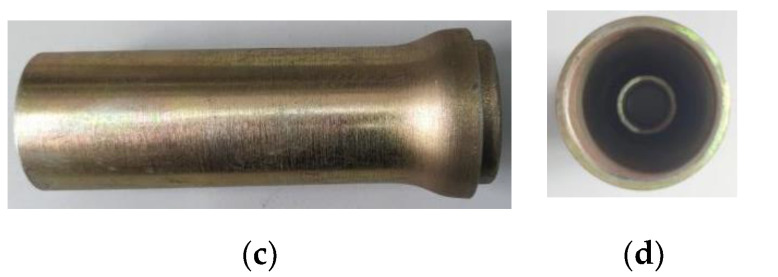
Constant resistance anti-impact device. (**a**) Flared thin-walled round tube, (**b**) special-shaped nut, (**c**) constant resistance anti-impact device combination chart, (**d**) top view of constant resistance anti-impact device.

Based on the above four basic features, the designed constant resistance anti-impact device is consisted by a flared thin-walled round tube and a special-shaped nut. The shaped nut has an aperture in the center with threads inside the aperture to connect to the right end of the rod with external threads. The inner diameter of the flared thin-walled circular tube orifice is larger than the diameter of the thin end of the special-shaped nut and smaller than the diameter of the thick end of the special-shaped nut. The inner diameter of the flared mouth is slightly larger than the diameter of the lower end of the profile nut, as shown in Figure 1.

### 2.2. The Composition of a Constant-Resistance Energy-Absorbing Anti-Scour Anchor

**Figure 2:**
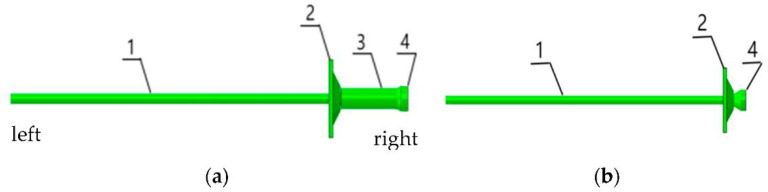
Anchor design drawings. (**a**) constant resistance energy-absorbing anti-scour anchor, (**b**) conventional anchors; 1: rod body; 2: tray; 3: anti-punch; 4: special-shaped nut.

Impact ground pressure roadway support anchors need to have instantaneous elongation and energy absorption under impact loads [30,31]; therefore, a constant resistance energy-absorbing anti-impact anchor rod was designed, consisting of a rod body, a tray, an anti-impact device and a shaped nut, with a shaped nut at the end of the anchor rod, and the anti-impact device is a thin-walled round tube with a flared end, which is connected to the shaped nut, and the anti-impact device is intermediate between the tray and the shaped nut. The relative position is shown in Figure 2a. Compared with the constant resistance energy-absorbing anchor rod, the conventional anchor rod consists of a rod body, a tray and a profile nut, and the relative positions are shown in Figure 2b.

The constant resistance deformation stage of constant resistance energy-absorbing anti-punch anchor is mainly a reflection of the deformation stage of the anti-puncher. Under pressure, the flared thin-walled round tube end and the thick end of the special-shaped nut are relatively misaligned to achieve an increase in the radius of the thin-walled round tube, and then in the downward compression process, the thin-walled round tube expansion area remains unchanged, i.e., in the compression process, the bearing capacity of the anti-puncher remains constant. See Section 3.2 for the specific deformation process

### 2.3. The Working Principle of a Constant Resistance Energy-Absorbing Anti-Scour Anchor

**Figure 3:**
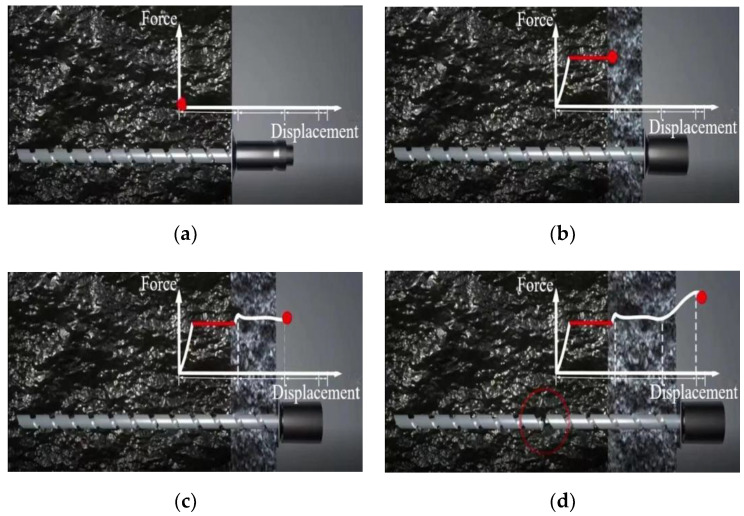
Deformation process of constant resistance energy-absorbing anti-scouring anchor rod by force, (**a**) initial stage (**b**) constant resistance deformation stage (**c**) yielding stage (**d**) strengthening and damage stage.

After the installation of the constant resistance energy-absorbing impact anchor (Figure 3a), under the impact load of the surrounding rock, when the impact load is less than the load bearing capacity of the constant resistance anti-impact device, the impact constant resistance anti-impact device does not deform, the rod is in the elastic stage and the load-bearing capacity rises linearly. When the impact load reaches the threshold value of the impact constant resistance anti-impact device deformation load, the constant resistance anti-impact device deforms and destroys, absorbing the impact energy during the deformation and destruction, the load-bearing capacity is at a constant stage (Figure 3b). After the deformation of the impact constant resistance anti-impact device ends, if the impact load is still greater than the threshold value of the constant resistance impact constant resistance anti-impact device deformation load, the rod becomes the loaded body and is pulled to the yielding stage, small increase in load capacity and constant (as in Figure 3c). If the rod yielding deformation stage ends, the impact load also continues to increase, the rod enters the strengthening stage and the carrying capacity continues to rise until the rod is pulled off and the anchor rod fails (as in Figure 3d).

The constant resistance deformation process of the anchor rod with constant resistance absorbs energy, which improves the force condition of the anchor rod under load and effectively improves the mechanical properties of the anchor rod. The constant resistance deformation stage of the anchor absorbs the deformation energy of the surrounding rock directly and also provides a certain distance for the deformation of the surrounding rock, which indirectly consumes part of the deformation energy of the surrounding rock, thus also improves the impact resistance of the support system of “support—surrounding rock” [32,33]. The effect of constant resistance energy-absorbing anchors and energy-absorbing brackets is better when used together.

## 3. Analysis of Mechanical Properties of an Anchor under Static Load

### 3.1. Model Building and Parameter Setting

The finite element model of the rod, pallet, energy absorber and shaped nut was established using the finite element software ABAQUS. The conventional anchor rod was 2500 mm long and 20 mm in diameter, the pallet size was 150 × 150 × 10 mm, the diameter of the central round hole was 22 mm, the shaped nut was 38 mm high, the diameter of the thin end was 28 mm, the diameter of the thick end was 48 mm, and the end taper angle was 26.5° (Figure 2a). The constant resistance energy-absorbing anchor rod is 2500 mm long and 20 mm in diameter; the shaped nut is 38 mm high, 28 mm in diameter at the thin end, 48 mm in diameter at the thick end, and the end taper angle is 26.5° [34]; the inner diameter of the anti-puncher is 40 mm, the wall thickness is 4 mm, and the height is 150 mm. The tray size is 150 × 150 × 10 mm, and the diameter of the central round hole is 22 mm (Figure 2b). The relative positions are assembled as shown in Figure 2, and the simulated tensioning process uses the power-explicit algorithm. The material parameters of the anchor, pallet, anti-puncher and shaped nut were set in the characteristics module. The density of the rod, pallet, energy-absorbing anti-puncher and shaped nut was 7850 kg/m^3^, the modulus of elasticity was 210 GPa, Poisson’s ratio was 0.3, and the plasticity parameters were taken from the data obtained from the laboratory anchor tensile test, and the yield strength of the material was 600 MPa and the tensile strength was 810 MPa after conversion. The rod was set up with flexible damage in ductile metal damage. The model’s boundary conditions are the left end of the rod is completely fixed, and only the axial displacement of the shaped nut end is allowed. A rigid plate with a diameter larger than the diameter of the rod with holes is used to displace 500 mm from the left side of the pallet to the right. in the mesh module, C3D8R cells are used for each component, the mesh shape is hexahedral, the pallet and rod mesh size are set to 5, the thin-walled circular tube mesh size is set to 2, and the cell type of the rod is set hourglass control for stiffness, and the cell is set to delete [35,36,37].

### 3.2. Comparative Analysis of Mechanical Properties with Conventional Anchor

**Figure 4:**
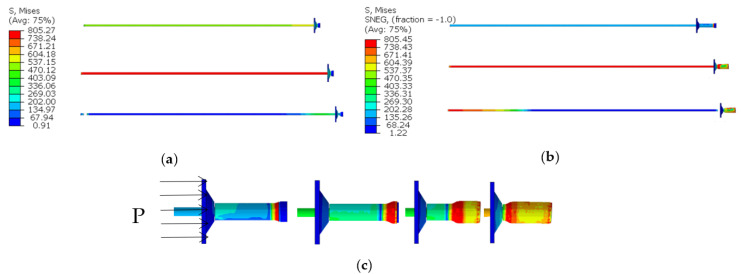
Anchor rod deformation by static load. (**a**) conventional anchors, (**b**) constant resistance energy-absorbing anti-scour anchor, (**c**) deformation of constant resistance anti-impact device by force.

**Figure 5:**
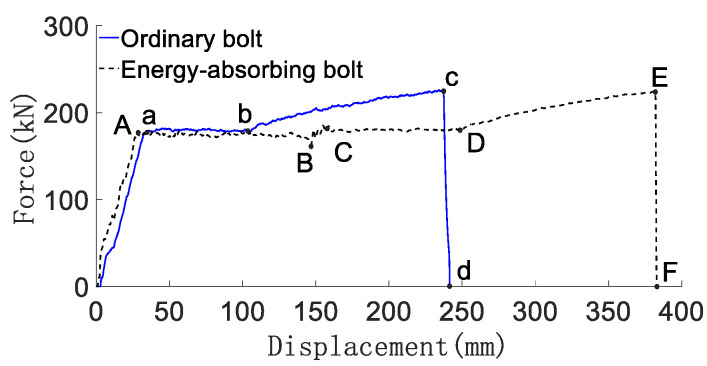
Anchor force-displacement curve.

The force deformation, force-displacement curve, energy absorption-displacement curve and mechanical properties of conventional anchor rods and constant resistance energy-absorbing anti-scour anchor rods are shown in Figure 4 and Figure 5, respectively, and are shown in Table 1.

From Figure 4, it can be obtained that the constant resistance energy-absorbing anti-scour anchor has a stable and repeatable deformation damage mode under static load.

**Figure 6:**
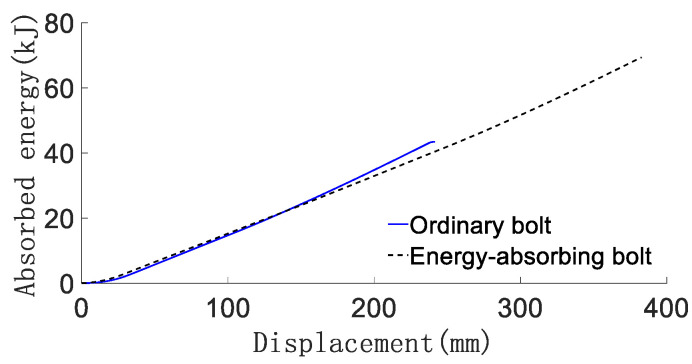
Anchor energy–absorption–displacement curve.

From Figure 5 and Figure 6 and Table 1, it can be obtained that: (1) the conventional anchor rod under load goes through the linear elastic phase (oA), yield phase, (ab), strengthening phase (bc), and damage phase (cd). The constant resistance energy-absorbing anti-strike anchor is loaded through the linear elastic phase (oA), the constant resistance deformation phase of the anti-strike (AB), the yield phase (CD), the strengthening phase (DE), and the damage phase (EF). Compared with the conventional anchor, the energy-absorbing impact-proof anchor adds the anti-impact constant-resistance deformation stage in the deformation process. (2) The constant resistance deformation distance of the constant resistance anti-impact device is about 135 mm, and the constant resistance deformation distance is 90% of the total length of the constant resistance anti-impact device, which indicates that the constant resistance anti-impact device has a high stroke efficiency. (3) The load-bearing capacity of the deformation stage of the anti-puncher is 160~165 kN, which means that the anti-puncher has a more constant load-bearing capacity. (4) The yielding distance of constant resistance energy-absorbing anti-charge anchor and conventional anchor are 375 mm and 240 mm, respectively, and the energy absorption is 66 kJ and 43 kJ, respectively. The yielding distance and energy absorption of constant resistance energy-absorbing anti-charge anchor are 1.53 times and 1.56 times of conventional anchor, respectively, which indicates that constant resistance energy-absorbing anti-charge anchor has strong mechanical properties of anti-charge.

## 4. Analysis of Mechanical Properties of Anchor under Impact Load

In order to derive the mechanical properties of energy-absorbing anti-shock anchor rods subjected to impact loads, ABAQUS software was used to numerically analyze the energy-absorbing anchor rods subjected to impact loads. Since the impact ground pressure releases a large amount of impact energy acting at the pallet of the anchor rod, a rigid body with mass and velocity is used to simulate the impact energy of the surrounding rock and to study the mechanical properties of the anchor rod under the action of different impact energy and impact velocity. The anchor rod parameters and static load are the same.

### 4.1. The Law of Impact Energy on The Mechanical Properties of Anchor

**Figure 7:**
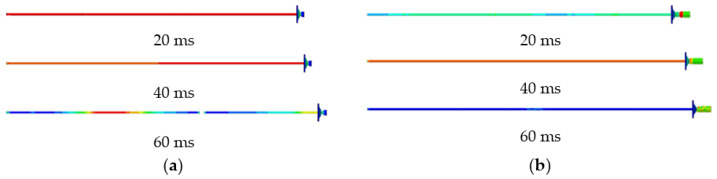
Deformation diagram of anchor rod subjected to impact load. (**a**) Conventional anchors, (**b**) constant resistance energy-absorbing anti-scour anchor.

**Figure 8:**
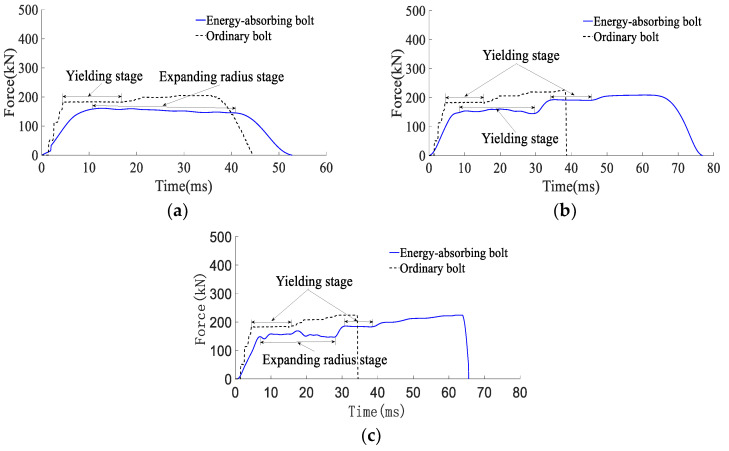
Anchor force–time curve: (**a**) impact energy 25 kJ; (**b**) impact energy 50 kJ; (**c**) impact energy 80 kJ.

**Figure 9:**
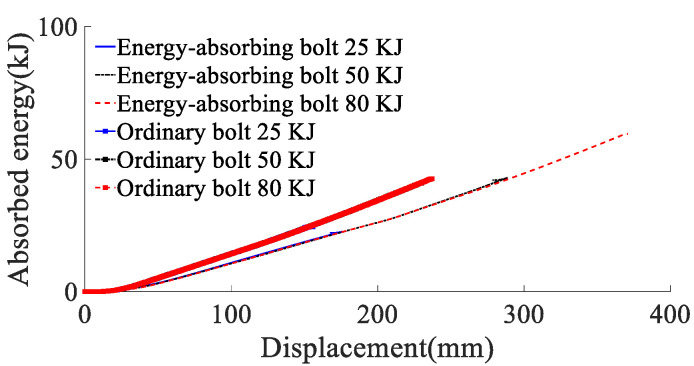
Anchor energy–absorption–displacement curve.

According to the energy absorption of anchor rod under static load, 25, 50 and 80 kJ perimeter rock impact energy (impact velocity is 8 m/s) is simulated to act on the anchor rod pallet, and the yield stress of the rod material is 600 MPa. The force deformation of conventional anchor rod and constant resistance energy absorption anchor rod under different impact energy is shown in Figure 7, the force-time curve is shown in Figure 8, the energy absorption-displacement curve is shown in Figure 9. The mechanical properties are shown in Table 2.

From Figure 7, the deformation stages experienced by both conventional and constant resistance energy-absorbing impact anchor rods under different impact energies are the same as those under static load. The constant resistance energy-absorbing impact anchor has a stable and repeatable deformation damage mode.

According to the results of the analysis of the mechanical properties of anchor rods under static load, combined with Figure 8 and Figure 9 and Table 2, it can be obtained that:

(1) When the impact energy of the surrounding rock is 25 kJ, both conventional anchor rod and constant resistance energy-absorbing impact anchor rod are not broken; when the impact energy of the surrounding rock is 50 kJ, the conventional anchor rod is broken and the constant resistance energy-absorbing impact anchor rod is not broken; when the impact energy of the surrounding rock is 80 kJ, both the conventional anchor rod and constant resistance energy-absorbing impact anchor rod are broken. It shows that the constant resistance energy-absorbing impact anchor has good mechanical properties of impact resistance;
(2) Under static load and 50 kJ and 80 kJ impact energy dynamic load, the yield load of the rod is 178, 183 and 183 kN, the breaking load is 226, 227 and 224 kN, the absorbed energy is 43, 41 and 43 kJ, and the yield distance is 240, 233 and 237 mm respectively. Compared with the static load, the yield load of the rod slightly increases, the yield distance slightly decreases, and the breaking load and absorbed energy remain the same. The effect of impact energy on the mechanical properties of conventional anchor rods can be ignored;
(3) Under static load and 80 kJ impact energy dynamic load, the yield load of the rod is 178 and 184 kN, the breaking load is 226 and 224 kN, the absorbed energy is 66 and 60 kJ, the yield distance is 375 and 370 mm, and the load capacity of the impact prevention device is 162 and 156 kN. Compared with the static load, the yield load of the rod increased slightly, the breaking load was basically unchanged, and the absorbed energy, the yield distance and the load carrying capacity of the anti-squeezer decreased by 9%, 3% and 4%, respectively;
(4) With the increase in impact energy, the bearing capacity of the deformation phase of the anti-puncher is 152~156 kN, which indicates that the anti-puncher has a more constant bearing capacity and deformation load threshold;
(5) When the impact energy of the surrounding rock is 80 kJ, the yield load of the rod is 183 and 184 kN, the breaking load is 228 and 227 kN, the impact resistance time is 35 and 65 ms, the absorbed energy is 43 and 60 kJ, and the yield distance is 229 and 370 mm for the conventional anchor rod and the constant resistance energy-absorbing impact anchor rod, respectively. The impact resistance time, absorbed energy and yield distance are 1.86, 1.40 and 1.61 times of conventional anchor rods, respectively, which means that the impact resistance mechanical performance of constant resistance energy-absorbing anchor rods is significantly better than that of conventional anchor rods.

### 4.2. The Law of Impact Velocity on The Mechanical Energy Absorption of Anchor

**Figure 10:**
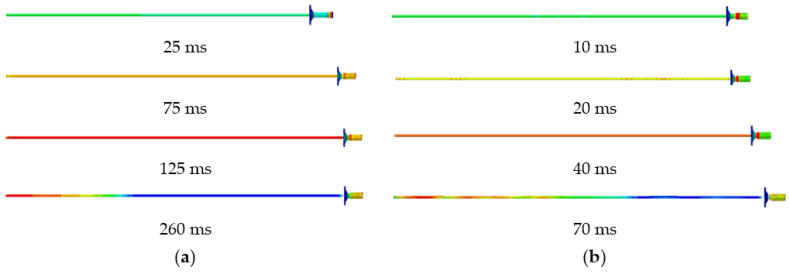
Deformation of constant resistance energy-absorbing anti-scouring anchor rod by force. (**a**) Conventional anchor, (**b**) constant resistance energy-absorbing anti-scour anchor.

**Figure 11:**
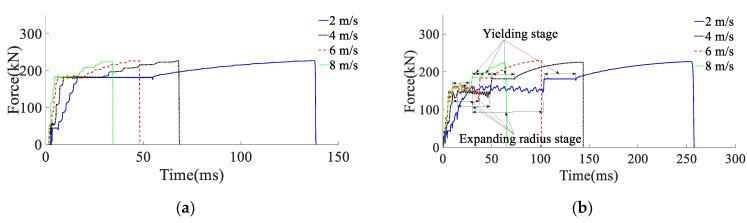
Anchor force–time curve. (**a**) Conventional anchor, (**b**) constant resistance energy-absorbing anti-scour anchor.

**Figure 12:**
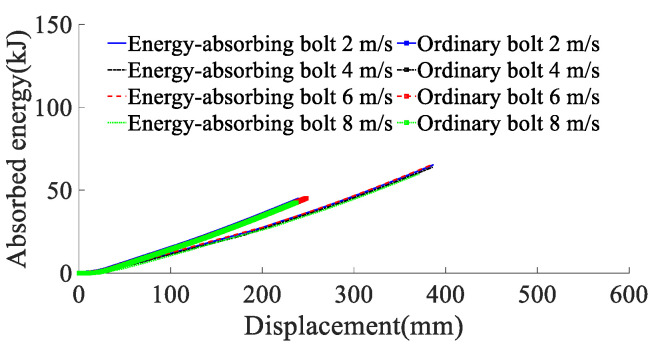
Anchor energy absorption–displacement curve.

**Figure 13:**
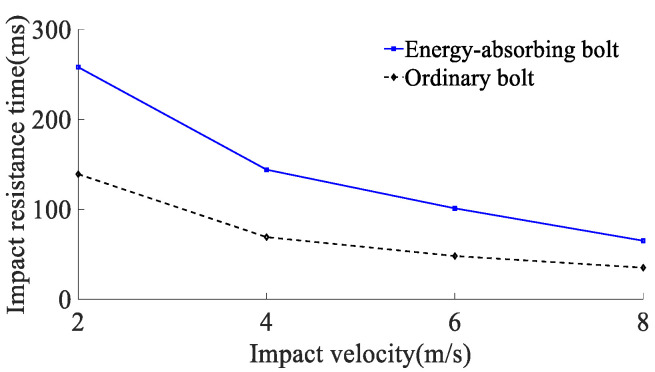
Anchor impact resistance time at different impact velocities.

**Figure 14:**
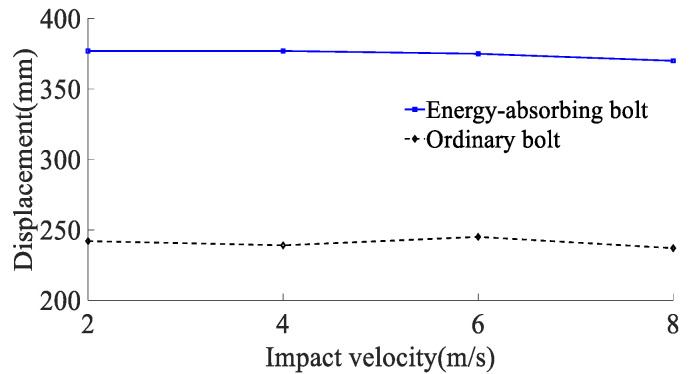
Anchor impact resistance time at different impact velocities.

According to the results of the analysis of the impact energy on the mechanical properties of anchor rods, the simulated impact velocity of the surrounding rock is 2, 4, 6, and 8 m/s (impact energy of the surrounding rock is 80 kJ) on the anchor rod tray, and the yield stress of the rod material is 600 MPa. The force deformation of conventional anchor rods and constant resistance energy-absorbing anchor rods at different impact velocities are shown in Figure 10, the force-time curve is shown in Figure 11, the absorbing energy-displacement curve is shown in Figure 12, and the mechanical properties are shown in Table 3. The mechanical properties are shown in Table 3. The impact resistance time of anchor rods at different impact velocities is shown in Figure 13, and the yield distance is shown in Figure 14.

According to the results of the analysis of the impact energy on the mechanical properties of anchor rods, the simulated impact velocity of the surrounding rock is 2, 4, 6, and 8 m/s (impact energy of the surrounding rock is 80 kJ) on the anchor rod tray, and the yield stress of the rod material is 600 MPa. The force deformation of conventional anchor rods and constant resistance energy-absorbing anchor rods at different impact velocities are shown in Figure 10, the force-time curve is shown in Figure 11, the absorbing energy-displacement curve is shown in Figure 12, and the mechanical properties are shown in Table 3. The mechanical properties are shown in Table 3. The impact resistance time of anchor rods at different impact velocities is shown in Figure 13, and the yield distance is shown in Figure 14.

From Figure 10, the deformation phases experienced by both conventional and constant resistance energy-absorbing anti-shock anchors during loading at different impact velocities are the same as those under static load, and the constant resistance energy-absorbing impact anchors have a stable and repeatable deformation damage mode.

From Figure 11, Figure 12, Figure 13 and Figure 14 and Table 2, it can be obtained that:

(1) At impact velocities of 2, 4, 6 and 8 m/s, the rod yield load is 181, 181, 182 and 183 kN, the breaking load is 228, 228, 227 and 224 kN, the absorbed energy is 44, 43, 44 and 43 kJ, the yield distance is 242, 239, 245 and 237 mm, and the impact resistance time is 139, 69, 48 and 35 ms. It shows that the impact velocity has a small effect on the rod yield load, breaking load, absorbed energy and yield distance of conventional anchor rods, and the impact time decreases non-linearly with the increase in impact velocity;
(2) When the impact velocity of constant resistance energy-absorbing anti-shock anchor is 2, 4, 6 and 8 m/s, the yield load of rod is 181, 181, 184 and 184 kN, the breaking load is 227, 226, 228 and 224 kN, the absorbed energy is 66, 64, 65 and 60 kJ, and the yield distance is 377, 377, 375 and 370 mm, respectively. The impact resistance time was 258, 144, 101 and 65 ms, respectively, indicating that the impact speed had a small effect on the rod yield load, breaking load, absorbed energy and yield distance of the constant resistance energy-absorbing anchor, and the impact resistance time decreased non-linearly with the increase in impact speed;
(3) The bearing capacity of the impact prevention device in the deformation phase at the impact speed of the surrounding rock is 2, 4, 6, and 8 m/s is 156, 149, 153, and 155 kN, the impact resistance time is 79, 39, 27, and 20 ms, and the yielding distance is 134, 132, 132, and 135 mm, indicating that the impact prevention device at different impact speeds has a more constant bearing capacity and a higher stroking efficiency;
(4) Under the same impact velocity, the three indexes of constant resistance energy-absorbing anti-impact anchor rods, such as yielding distance, impact resistance time and energy absorption, are significantly better than those of conventional anchor rods.

## 5. Field Applications

### 5.1. Site Overview

**Figure 15:**
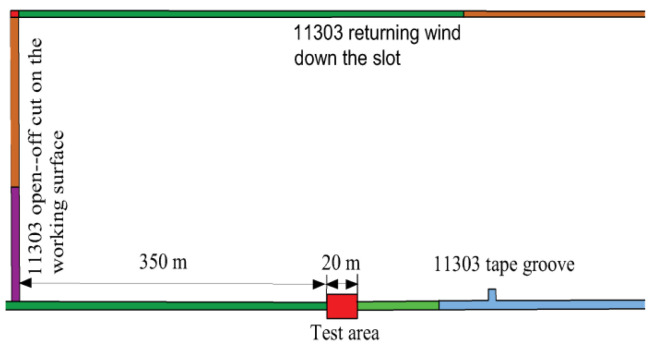
Test area of 11303 belt chute.

The Hongqingliang coal mine belongs to the impact pressure mine, and a 11303 tape chute, which is a concentrated stress area in the Hongqingliang coal mine, was selected for the field application test, and the test area was within 350–370 m from the 11303 working face cuthole, as shown in Figure 15. The test area is about 20 m, and the roadway is supported by anchor rod and anchor cable.

**Figure 16:**
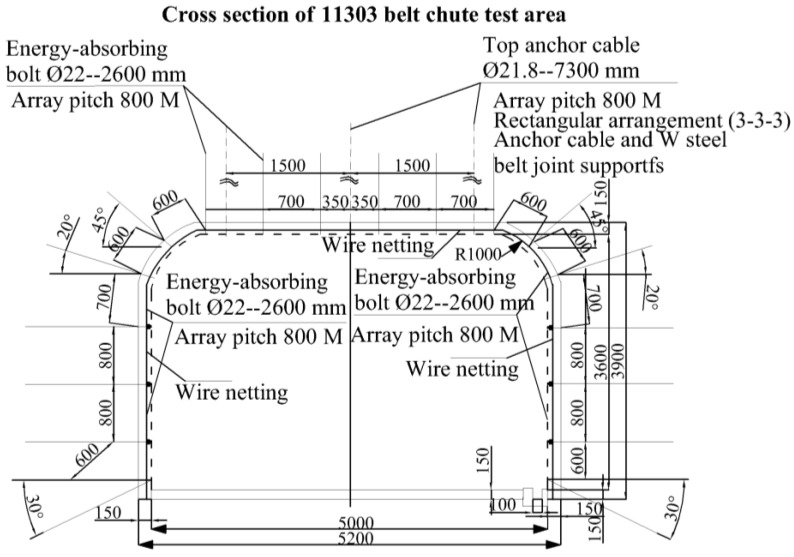
Section of support in the test area of 11303 tape chute.

The anchor rods are 2600 mm in length, 22 mm in diameter and 150 mm in length, and are used in conjunction with the metal mesh in the gang section of the roadway, with 800 mm anchor rows in the gang section and 700 × 1000 mm rows in the roof section, while the anchor ropes are φ21.6 × 7300 mm prestressed anchor ropes with a 3–3–3 arrangement and 2000 mm rows. The situation is shown in Figure 16.

### 5.2. Field Test Results

**Figure 17:**
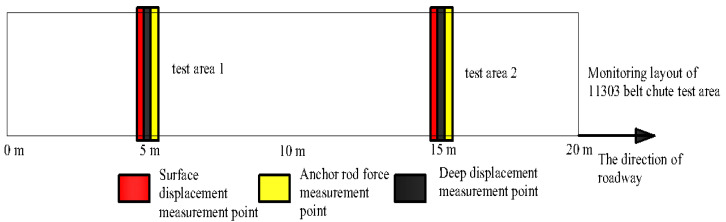
Monitoring layout of 11303 belt chute test area.

**Figure 18:**
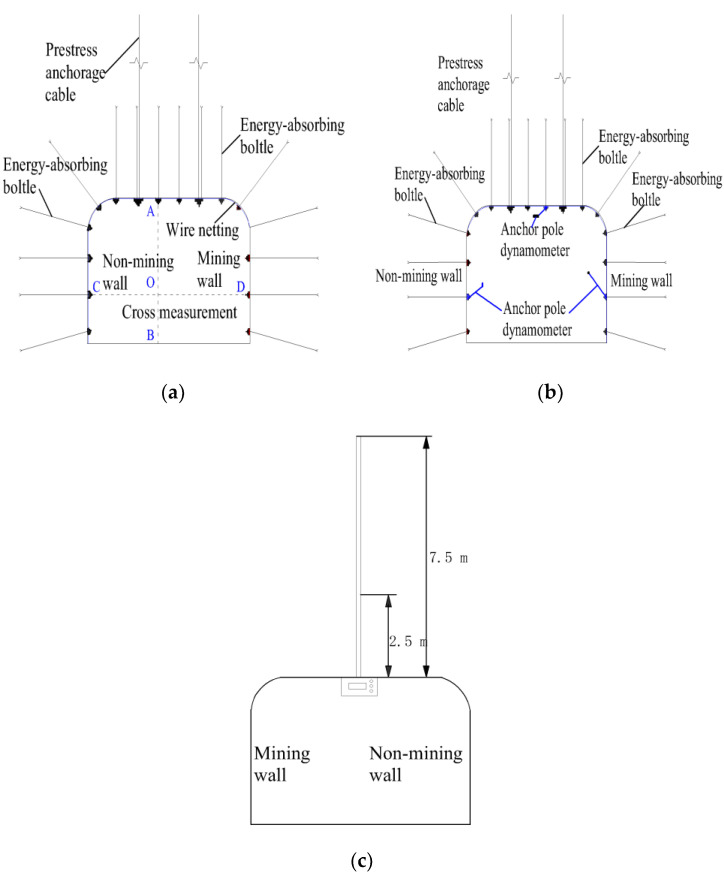
Specific monitoring scheme of 11303 belt chute monitoring area. (**a**) Surface displacement monitoring; (**b**) anchor force monitoring. (**c**) deep displacement monitoring.

The test area of 11303 tape chute is subjected to macro mine pressure observation, surface displacement monitoring, deep displacement monitoring and anchor rod force monitoring, etc. The monitoring plan is as follows: 2 measurement areas are arranged in the test area, 10 m apart, surface displacement monitoring points are monitored every 3 days; deep displacement measurement points and anchor rod force measurement points are monitored online. The monitoring points and layout of the roadway are shown in Figure 17 and Figure 18.

The results of mine pressure monitoring in the test area of 11303 tape chute show that the change of surface displacement is not obvious, the overall top sinkage is small, and the maximum top sinkage is less than 25 mm; the bulging of the sides are asymmetric, and the two sides are more obvious than the non-mining sides, but none of them exceed 60 mm. Through online analysis, it is found that the anchor force on the top slab increases significantly by about 35 kN; the anchor force on the wall part is uniform and does not show any obvious trend of increase; the deep displacement measurement points do not change significantly. Through repeated roadway observation, the roadway integrity in the test area of 11303 tape chute is good, no obvious roadway top sink, bottom drum and other mineral pressure phenomenon, after using the new energy-absorbing anti-punching anchor, no anchor failure phenomenon such as anchor rod breakage. The overall support effect is good. The power phenomenon has not appeared during the support time, and the impact resistance has to be further verified on site.

## 6. Conclusions

(1) Based on the requirements that the anchor rod anti-scouring device should have a reasonable deformation load threshold, high stroke efficiency, a constant reaction force and stable repeatable deformation damage mode, a constant resistance anti-scouring device is designed. Additionally, a constant-resistance energy-absorbing anti-stroke anchor rod consisting of rod body, tray, anti-stroke and profiled nut is designed, and the working principle of constant-resistance energy-absorbing anti-stroke anchor rod is given;
(2) The constant resistance energy-absorbing anti-stroke anchor has a stable and repeatable deformation damage mode under both static and impact loads. The impact energy and impact velocity have less influence on the load carrying capacity and stroke efficiency of the constant resistance anti-impact device;
(3) The impact velocity has a small effect on the indices of rod yield load, breaking load, absorbed energy and yield distance of conventional anchor rods and constant resistance energy-absorbing anti-shock anchor rods, and the impact resistance time all decreases non-linearly with the increase in impact velocity;
(4) Under static and impact loads, the three indexes of constant resistance energy-absorbing anti-shock anchor rods, such as yielding distance, impact resistance time and energy absorption, are significantly better than those of conventional anchor rods.

## Figures and Tables

**Table 1:** Comparison of anchor force characteristics.

Anchor Rods Type	Yield Load/kN	Breaking Load/kN	Energy Absorption/kJ	Letting Distance/mm	Anti-Scouring Device Bearing Capacity/kN
Conventional anchors	178	226	43	240	
Constant resistance energy-absorbing anti-scour anchors	178	226	66	375	160–165

**Table 2:** Mechanical properties of anchor rods at different impact energies.

Anchor Rods Type	Impact Energy/kJ	Yield Load/kN	Breaking Load/kN	Anti-Impact Time/ms	Energy Absorption/kJ	Letting Distance/mm	Anti-Scouring Device Bearing Capacity/kN
Conventional anchors	25	182	-	44	24	155	-
Conventional anchors	50	183	227	39	41	233	-
Conventional anchors	80	183	224	35	43	237	-
Constant resistance energy-absorbing anti-scour anchor rods	25	-	-	53	23	176	Approx. 152
Constant resistance energy-absorbing anti-scour anchor rods	50	183	-	77	43	289	Approximately 156
Constant resistance energy-absorbing anti-scour anchor rods	80	184	224	65	60	370	Approximately 156

**Table 3:** Mechanical properties of anchor rods at different impact velocities.

Anchor Type	Impact Speed/m/s	Yield Load/kN	Breaking Load/kN	Antiflush Time/ms	Rod Impact Resistance Time/ms	Energy Absorption/kJ	Anti-Puncher Give Way Distance/mm	The Distance of the Rod Gives Way/mm	Anti-Scouring Device Bearing Capacity/kN
Conventional anchors	2	181	228	-	139	44	-	242	-
Conventional anchors	4	181	228	-	69	43	-	239	-
Conventional anchors	6	182	227	-	48	44	-	245	-
Conventional anchors	8	183	224	-	35	43	-	237	-
Constant resistance energy-absorbing anti-scour anchor rods	2	181	227	79	179	66	134	243	Approximately 156
Constant resistance energy-absorbing anti-scour anchor rods	4	181	226	39	105	64	132	245	Approximately 149
Constant resistance energy-absorbing anti-scour anchor rods	6	184	228	27	74	65	132	243	Approximately 153
Constant resistance energy-absorbing anti-scour anchor rods	8	184	224	20	45	60	135	235	Approximately 155

## Data Availability

Not applicable.
